# Barrett's Esophagus in Rubinstein-Taybi Syndrome

**DOI:** 10.7759/cureus.11709

**Published:** 2020-11-25

**Authors:** Prabhat Kumar, Prashanthi N Thota

**Affiliations:** 1 Gastroenterology, Cleveland Clinic, Cleveland, USA

**Keywords:** barrett's esophagus, rubinstein-taybi syndrome, low-grade dysplasia, gastroesophageal reflux disease, esophageal stricture, dysphagia

## Abstract

Rubinstein-Taybi syndrome (RSTS; Online Mendelian Inheritance in Man^® ^[OMIM^®^] #180849, #613684; Orpha: 783 ) is a rare plurimalformative autosomal dominant genetic disorder that affects one in 100,000-125,000 newborns with equal male and female distribution. It is characterized by distinctive facial features, short stature, broad and often angulated thumbs and halluces, and moderate-to-severe intellectual disability. In addition to ocular, cardiac, renal, endocrinologic, neurological, and psychomotor abnormalities, RSTS individuals can present with several gastrointestinal symptoms such as feeding difficulties, gastroesophageal reflux, and constipation. Currently, therapeutic strategies for RSTS involves a multi-disciplinary approach focusing mainly on symptomatic management. Here, we present a case of young-onset Barrett's esophagus in a patient with Rubinstein-Taybi syndrome.

## Introduction

Rubinstein-Taybi syndrome (RSTS) is a rare plurimalformative genetic disorder first described in 1963 by JH Rubinstein and H Taybi [1]. RSTS, also known as the broad thumb-hallux syndrome, is an autosomal dominant disease that affects one in 100,000-125,000 newborns with an equal male:female distribution [2]. It is characterized by distinctive facial features (hypertelorism, microcephaly, protruding beaked nose, downward-slanted eyes, thick and arched eyebrows), talon cusps teeth, short stature, broad and often angulated thumbs and halluces, and moderate-to-severe intellectual disability [3]. The differential diagnosis may include the Genitopatellar syndrome, the Floating-Harbour syndrome, and the Cornelia de Lange syndrome.

There are two types of RSTS. RSTS type 1 is caused by submicroscopic deletion of the cAMP response element-binding protein gene (CREBBP) located on the 156-kb region on chromosome 16p13.3 (50%-60%), and type 2 is caused by a mutation in the EP300 gene on chromosome 22q13.2 (~8%) [4]. However, in some people with RSTS, the cause is unknown. RSTS diagnosis is predominantly based on a physical examination and genetic testing by fluorescence in situ hybridization and genetic sequencing. Family studies and genetic studies suggest that the risk of recurrence of RSTS in siblings is less than one percent.

Gastrointestinal symptoms such as feeding difficulties, gastroesophageal reflux (GERD) (68%), constipation (40-74%), and Hirschsprung disease are prevalent in these patients [3]. If not promptly recognized and managed, these symptoms can lead to secondary complications such as failure to thrive and esophageal strictures.

## Case presentation

**Figure 1:**
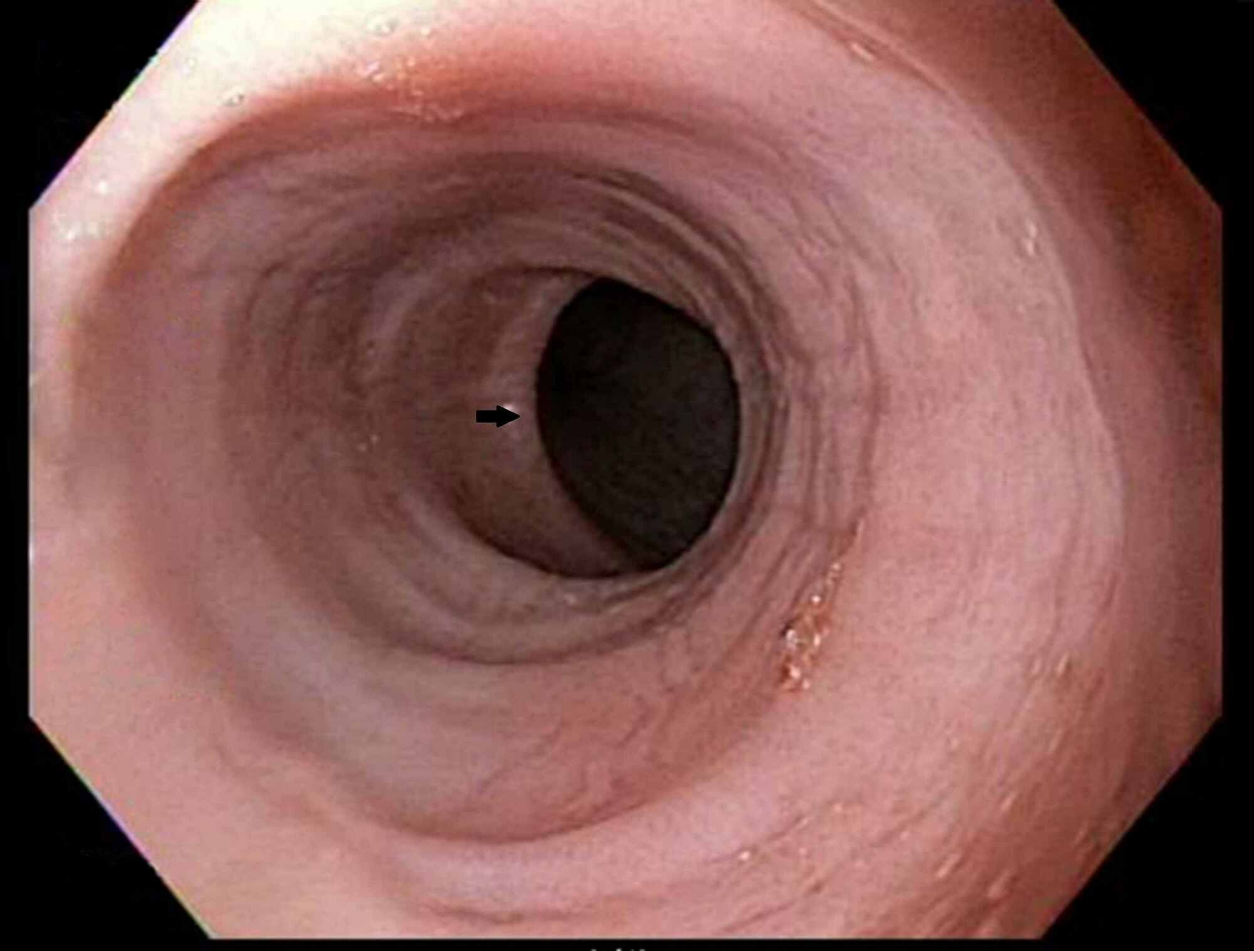
Esophageal stricture

**Figure 2:**
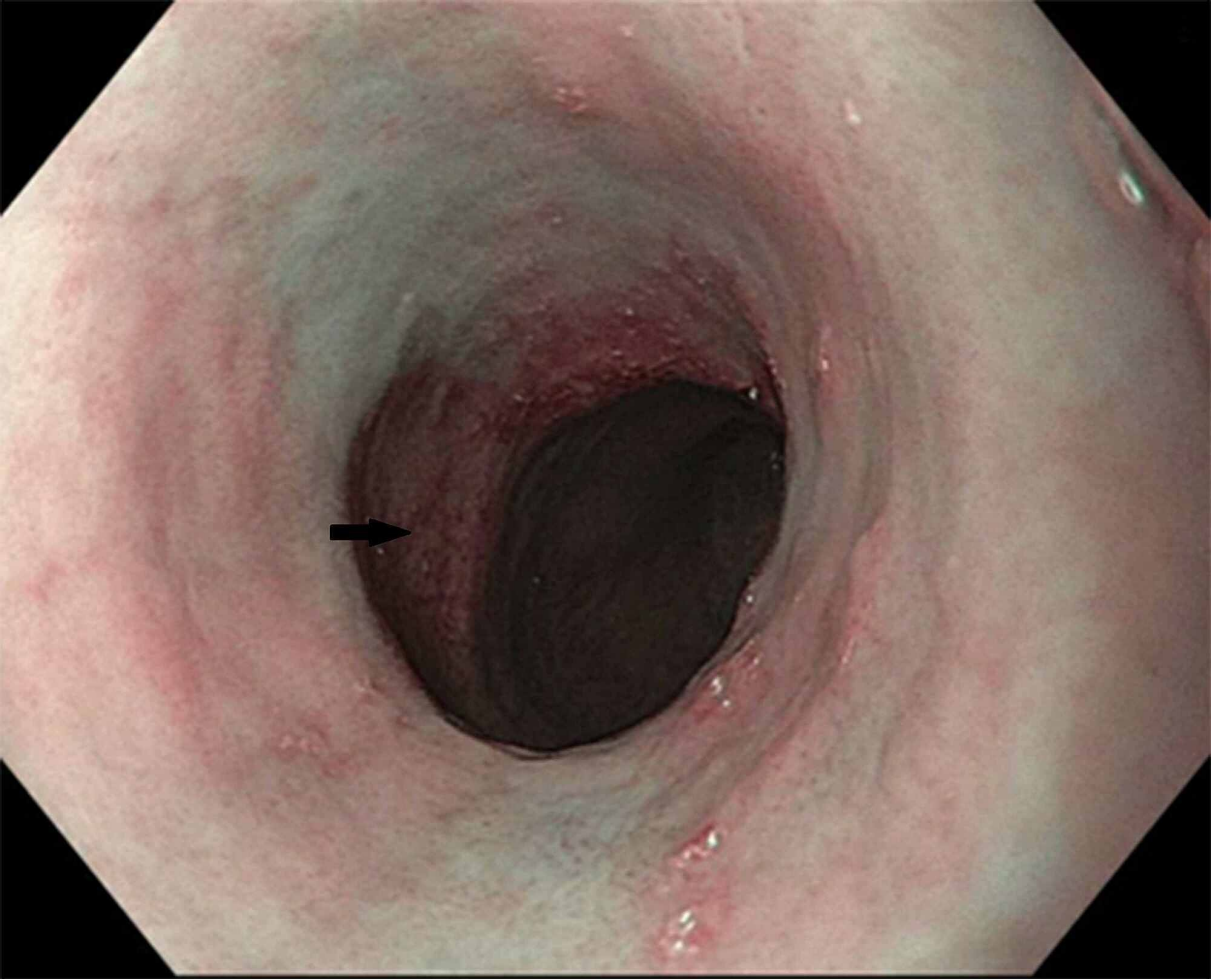
Barrett's esophagus

A 26-year-old non-Hispanic white man diagnosed with RSTS presented with dysphagia to solids and was found to have a cervical web on the esophagram. Esophagogastroduodenoscopy (EGD) showed an esophageal stricture extending from 18 to 22 cm, which was treated by endoscopic balloon dilation (Figure 1). A long-segment Barrett's esophagus (BE) extending from 22 to 32 cm was also present (Figure 2). Biopsies showed intestinal metaplasia without any dysplasia. The patient was treated with high dose proton pump inhibitor therapy twice a day. Since then, he was on surveillance for BE and underwent periodic dilations for esophageal stricture. Twelve years after the initial diagnosis of BE, he was found to low-grade dysplasia within the BE segment during periodic surveillance. He underwent one session of radiofrequency ablation therapy after stricture dilation. A follow-up EGD showed a 1 cm segment of BE with complete eradication of dysplasia. Two years later, he presented with recurrent dysphagia. EGD revealed a 5 cm long proximal esophageal stricture, which was dilated with an endoscopic balloon. There was also a 3 cm long BE segment in the distal esophagus with biopsies revealing low-grade dysplasia. The patient was scheduled for a repeat ablation session. 

## Discussion

RSTS is associated with several gastrointestinal manifestations such as GERD, dysphagia, constipation, and rarely, Hirschsprung disease [3, 5]. Several case reports have illustrated various esophageal disorders such as GERD presenting with recurrent respiratory problems, post cricoid webs, eosinophilic esophagitis, and mediastinal vascular rings [5-8]. 

Here, we report the first case of young-onset BE in a patient with RSTS. BE is a premalignant condition characterized by replacing more than 1 cm of normal squamous epithelium with specialized intestinal metaplasia in the distal esophagus that often develops as an adaptive response to chronic GERD. The risk factors for BE include Caucasian race, male gender, age >50, severe GERD symptoms for more than five years, smoking, hiatal hernia, and family history of BE or esophageal adenocarcinoma (EAC) [9].

What is unusual in this case is the young age at the diagnosis of BE. Our patient has chronic GERD symptoms for more than 15 years before BE diagnosis. A review of prior literature shows that the prevalence of GERD among institutionalized individuals with an intellectual disability is about 50%, with 70% of these GERD patients having reflux esophagitis and 14% having BE. GERD has also been shown to be associated with cerebral palsy, an intelligence quotient (IQ) < 35, scoliosis, and the use of anticonvulsant drugs or benzodiazepines. To establish the diagnosis, 24-h pH measurement, or EGD, should be used in intellectually disabled individuals in whom GERD clinically is suspected [10]. Individuals with RSTS have varying degrees of intellectual disability; hence these individuals should be carefully screened to prevent any progression to GERD complications such as BE and EAC. Once diagnosed with BE, they should undergo periodic surveillance as per guidelines [9].

## Conclusions

Since RSTS individuals can present with GERD symptoms and have a varying degree of intellectual disability, physicians should be vigilant about the risk of complications of GERD, such as esophageal strictures and BE. Hence, a prompt evaluation with EGD is indicated when these patients present with esophageal symptoms. Future studies are needed to assess the risk of EAC in these patients.
